# Alterations of Blood Pressure and ECG following Two-Week Consumption of *Berberis integerrima* Fruit Extract

**DOI:** 10.1155/2014/209683

**Published:** 2014-10-29

**Authors:** Siyavash Joukar, Naser Mahdavi

**Affiliations:** ^1^Physiology Research Center, Institute of Neuropharmacology, Kerman University of Medical Sciences, Kerman, Iran; ^2^Department of Physiology and Pharmacology, School of Medicine, Kerman University of Medical Sciences, P.O. Box 7616914115, Kerman, Iran

## Abstract

In light of the popularity and also the various nutritional and medicinal properties of *Berberis integerrima*, this study was conducted to assess the influence of its aqueous extract on hemodynamic and electrocardiogram (ECG) indices of rat. Animals were divided to control (CTL), B50, B100, and B200 groups that orally received tap water, aqueous extracts of *B. integerrima* fruit 50, 100, and 200 mg/kg/day, respectively, for two weeks and on day 15, data were recorded. Different doses of barberry fruit extract had no significant effect on blood pressure, heart rate, RR interval, P duration, and Q wave amplitude of electrocardiogram. Extract administration was associated with an incremental trend in PR interval that was not statistically significant. Higher doses (100 and 200 mg/kg) of extract significantly increased the QRS interval (*P* < 0.01 versus CTL and B50 groups) but decreased the QTc interval (*P* < 0.01 versus CTL group and *P* < 0.001 versus B50 group), the JT interval, and TpTe interval (*P* < 0.001 versus CTL and B50 groups). The results suggest that high doses of barberry extract definitely prolong the depolarization phase and shorten the repolarization phase of ventricular muscle and hence induce alteration in heart electrical conductivity.

## 1. Introduction

Different species of barberry (Zereshk in Persian) grows and is well known in different parts of the world such as Iran, China, middle Asia, and many countries in Europe, Africa, and America. Various parts of this plant including its root, bark, leaf, and fruit have been used as folk medicine for long time in Iran and other communities [1, 2]. Barberry fruits are used as a garniture in Persian food and in preparing juices, flake, honey, sauces, jellies, carbonated drinks, candy, food color powder, jam, marmalade, chocolates, and fruit nectars [3]. In Iranian traditional medicine several properties such as antibacterial, antipyretic, antipruritic, antihypertensive, and antiarrhythmic effects for different parts of barberry have been reported [4]. It has been shown that the crude extract of barberry has antihistaminic and anticholinergic activities [1]. In addition, recently, hypoglycemic, hypolipidemic, and antioxidant effects of this plant are reported in diabetic patients and rats [5, 6].

Two famous barberry species* B. integerrima* (abi) and* B. vulgaris* (poloei) are in Iran.* B. integerrima* is a thorny shrub with fragile branches to a height of 1 to 3 meters. Its red and oval fruits are 7–10 mm long and 3-4 mm in diameter, with a mild sour taste [3]. Major chemical compounds of barberry fruits include Coumarin, Ascorbic acid, Berberine, Caffeic acid, beta Carotene, Flavonoids, Malic acid, Palmatine, Carbohydrates, Tannin, and Ursolic acid [7]. Overall, amounts of ash, fiber, protein, reducing sugars, total sugar, and pH are higher in* B. integerrima* whereas moisture content, brix, and colour indices are higher in* B. vulgaris*. The amounts of P, Zn, Fe, Na, and K in* B. vulgaris* are higher than those of* B. integerrima* and the amounts of Mn, Mg, and Cu, Ca are higher in fruits of* B. integerrima*. In addition total phenolic and total anthocyanin contents of fresh fruits of* B. integerrima* are higher than fresh fruits of* B. vulgaris* [3].

Studies show that the alkaloid constituents with an isoquinolinic nucleus such as berberine, berbamine, and palmatine are important compounds of this plant [8] and of these berberine is the most important and predominant. Berberine is generally claimed to be only responsible for beneficial effects of the plant and so far numerous studies have been conducted on it. However it is suggested that other alkaloids and compounds also have some roles in the therapeutic effects of the plant [9].

There are numerous substances and drugs that can cause electrocardiogram (ECG) changes, even in patients without history of cardiac pathology. It is clear that the ECG is an easy, fast, and valuable tool to evaluate the safety and the positive or negative effects of cardiovascular drugs on heart electrical properties and conductivity. However, many drugs with no cardiovascular indication or with no overt cardiovascular effects from therapeutic dosing become cardiotoxic in overdose [10]. For example, there is a significant association between prolonged ventricular repolarization (measured as the corrected QT interval) and risk of sudden cardiac death (SCD) [11].

Despite the common use of barberry fruits in many communities particularly in the Middle East and global popularity in Iran and also discovery of new pharmacological properties of this fruit in recent years, the lack of adequate information regarding its effects on cardiac electrophysiology in literature is obvious. Hence, the present study was designed to investigate the effect of* Berberis integerrima* fruit consumption on electrocardiogram parameters of rat.

## 2. Material and Method

The study was conducted in accordance with the guidelines of animal studies provided by Ethics Committee Permission number 90/315, Kerman University of Medical Sciences, and performed on 30 male Wistar rats weighing 250–300 g, purchased from the laboratory animal breeding center of medical school, Kerman University of Medical Sciences, Kerman, Iran. Animals were housed in a temperature-controlled room and were allowed free access to rat regular diet and water.

### 2.1. Preparation of Barberry Extract

*Berberis integerrima* fruits were collected from Baft area of Kerman, Iran, and identified and a voucher specimen (KF-1194) of the plant was deposited in the Herbarium Center, Faculty of Pharmacy, Kerman University of Medical Sciences. After grinding, every 10 gr of barberries was boiled with 100 gr distillated water for 5 minutes. The filtered aqueous extract was concentrated in a rotary vacuum evaporator and dried by exposure to hot air to give solid material. Then, barberry extracts were kept in glass vials at −20°C prior to and during use [4].

### 2.2. Experimental Protocol

Animals were randomly divided to 4 groups. The control group (CTL) received 1 cc/kg of tap water by gavage method for 14 days. Barberry groups including B50, B100, and B200 were treated with barberry extract by gavage with doses of 50, 100, and 200 mg/kg/day, respectively, for two weeks. On day 15, animals were deeply anesthetized by intraperitoneal injection of sodium thiopental (50 mg/kg, i.p.). Animals had spontaneously breathing throughout the experiment. The left common carotid artery was cannulated by a filled catheter (saline with 15 IU/mL heparin) and connected to a pressure transducer of PowerLab system and arterial blood pressure (BP) was recorded during the experiment [12].

The ECG was recorded with standard limb lead II using a PowerLab data acquisition system and the duration and voltage of waves and also intervals of ECG were determined by means of 5 min ECG recorded strip. The PR interval embraces both atrial activation (P wave) and the time for conduction through the atrioventricular node and the specialized His-Purkinje system to the ventricles. PR interval was measured from the earliest P-wave onset to the earliest onset of the QRS complex (Q- or R-wave) onset [13]. The QT interval encompasses both depolarization and repolarization of heart and was measured from the earliest Q- or R-wave onset to the end of T wave. In order to obviate the dependence of QT interval on heart rate, corrected QT (QTc) intervals were measured using Bazett's formula normalized as QTcn-B = QT/(RR/*f*)^1/2^, where RR is R-R interval and *f* = 150 ms [14, 15].

### 2.3. Statistical Analysis

The data are presented as the mean ± SEM. Comparisons were performed among different groups by one-way ANOVA and post hoc Tukey's test. *P* values less than 0.05 were considered as statistically significant.

## 3. Result

**Figure 1:**
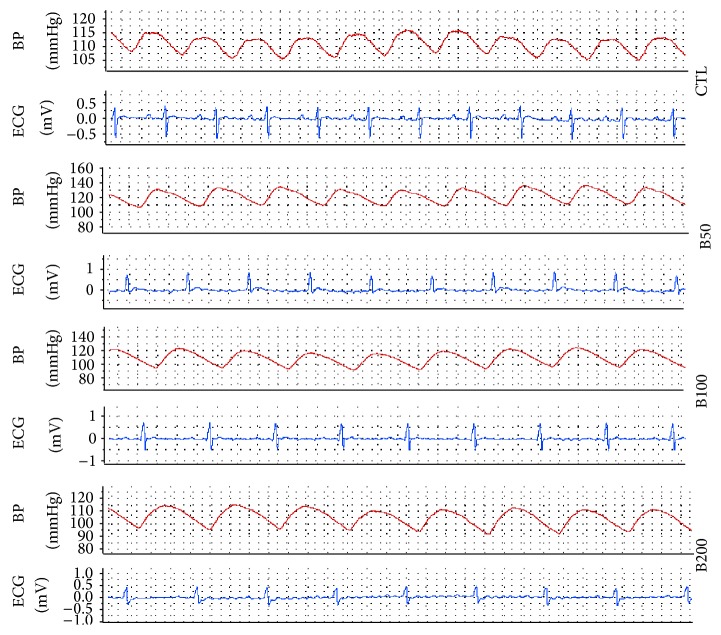
The strips of arterial blood pressure and lead II of ECG that simultaneously was recorded from an animal in each group. CTL: the control group, B50: animal group which received 50 mg/kg/day of barberry extract, B100: animal group which received 100 mg/kg/day of barberry extract, and B200: animal group which received 200 mg/kg/day of barberry extract.

Figure 1 shows a trace of arterial blood pressure and lead II of ECG that simultaneously was recorded from a rat of each animal group.

**Figure 2:**
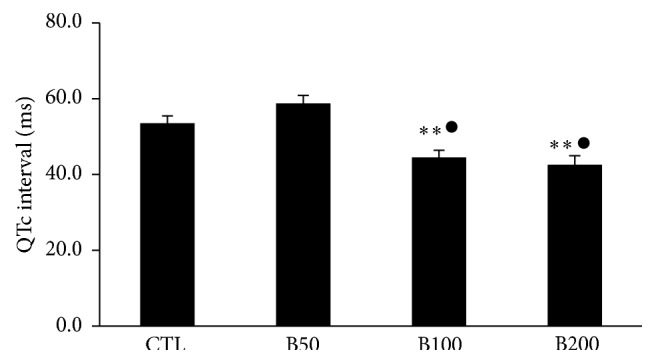
The corrected QT interval in animals groups. Values are expressed in mean ± SEM, CTL: the control group, B50: animal group which received 50 mg/kg/day of barberry extract, B100: animal group which received 100 mg/kg/day of barberry extract, and B200: animal group which received 200 mg/kg/day of barberry extract, *n* = 7-8. ^∗∗^ *P* < 0.01 versus CTL group and ^●^ *P* < 0.001 versus B50 group.

**Figure 3:**
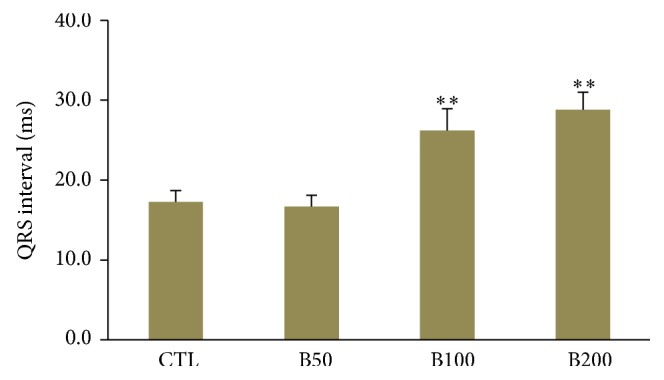
The QRS interval in all groups. Values are expressed in mean ± SEM, CTL: the control group, B50: animal group which received 50 mg/kg/day of barberry extract, B100: animal group which received 100 mg/kg/day of barberry extract, and B200: animal group which received 200 mg/kg/day of barberry extract, *n* = 7-8. ^∗∗^ *P* < 0.01 versus CTL and B50 groups.

**Figure 4:**
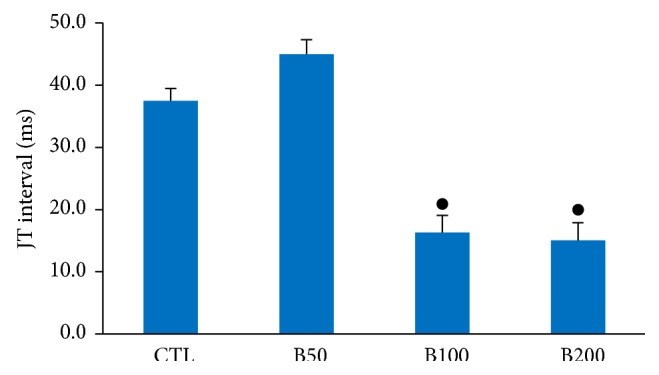
The JT interval in animals groups. Values are expressed in mean ± SEM, CTL: the control group, B50: animal group which received 50 mg/kg/day of barberry extract, B100: animal group which received 100 mg/kg/day of barberry extract, and B200: animal group which received 200 mg/kg/day of barberry extract, *n* = 7-8. ^●^ *P* ≤ 0.001 versus B50 and CTL groups.

**Figure 5:**
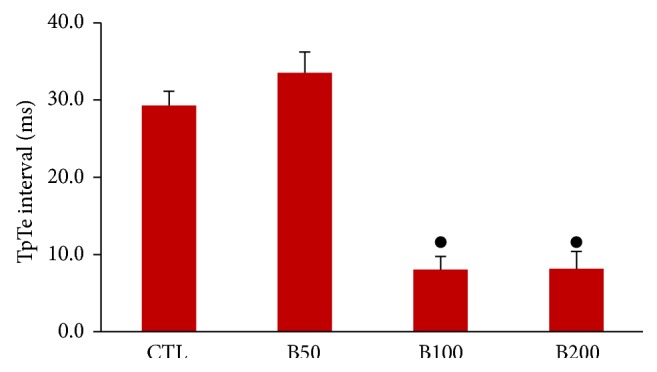
The TpTe interval in all groups. Values are expressed in mean ± SEM, CTL: the control group; B50: barberry extract with dose of 50 mg/kg; B100: barberry extract with dose of 100 mg/kg; B200: barberry extract with dose of 200 mg/kg; *n* = 7-8. ^●^ *P* ≤ 0.001 versus B50 and CTL groups.

Two-week consumption of different doses of* Berberis integerrima* fruits extract did not induce significant alteration in heart rate and also RR interval, P duration, and Q amplitude of ECG in rat. The PR interval showed an incremental trend in presence of barberry consumption but was not statistically significant (Table 1). Administration of barberry extract with dose 50 mg/kg had no significant effect on QTc interval whenever compared with control group. However the doses of 100 mg/kg and 200 mg/kg significantly reduced the QTc interval compared to control group (*P* < 0.01 and *P* ≤ 0.001, resp.) (Figure 2). Consumption of barberry extract with doses of 100 and 200 (mg/kg) significantly prolonged QRS interval compared to control group (*P* < 0.01 and *P* ≤ 0.001, resp.) (Figure 3). In addition, doses of 100 and 200 (mg/kg) of extract significantly decreased JT and TpTe intervals when compared with control and B50 groups (*P* < 0.001) (Figures 4 and 5).

## 4. Discussion

In present study barberry extract with doses of 100 and 200 (mg/kg), unlike dose 50 mg/kg, significantly decreased the QTc, TpTe, and JT intervals compared to control group. However, the QRS interval was prolonged in B100 and B200 groups versus control group.

The QRS complex represents activation of ventricular myocardium, and QRS prolongation may result from a wide range of processes involving the conduction system primarily, the myocardium, or both. QRS prolongation may involve either diffuse processes such as hypertrophic or infiltrative diseases, or drugs, or focal damage from infarction or infiltration [13]. Sodium channel blockers including class I antiarrhythmics by blocking inward sodium current can slow depolarization, reduce conduction velocity, and prolong the QRS interval and consequently may increase the risk of arrhythmia [13, 16]. Based on QRS interval prolongation induced by high doses of barberry fruit, it is possible that barberry can slow ventricular conductivity through the blockade of sodium current. The fast sodium channels are responsible for the rapid depolarization (phase 0) of fast-response cardiac action potentials. Blocking these channels decreases the slope of phase 0, which also leads to a decrease in the amplitude of the action potential and hence slows the ventricular conduction [17]. Regardless of involving mechanisms, similar to the effect of Class I antiarrhythmic drugs, this effect could potentially be antiarrhythmic and in high doses of extract could be arrhythmogenesis.

The QT interval is equal to the depolarization plus repolarization periods of the heart. JT interval is interval in an electrocardiogram from junction point J to T wave end and describes the duration of ventricular repolarization [18]. Tpeak to Tend interval (TpTe), the interval from the peak of the T wave to the end of the T wave, is a mark of transmural dissemination of repolarization in the left ventricle [19, 20]. Prolongation of these intervals increases the potential vulnerability to reentry ventricular arrhythmias [21, 22] and risk of sudden cardiac death [23, 24]. There are three cell types identified in the ventricular myocardium: the endocardial, epicardial, and subendocardial M cells (Masonic mid-myocardial Moe cells) [25, 26]. During bradycardia, the action potential of the M cells is more vulnerable to prolongation compared to the other two cell types [20], likely due to larger late sodium and sodium/calcium exchange currents and a weaker slowly activating delayed rectifier current (IKs) [27]. In period of TpTe interval during which the epicardium has repolarized, the M cells are still in the process of repolarization and vulnerable to the occurrence of early after-depolarizations (EADs) [28] and in turn it can lead to reentry and VT or VF. Hence, a prolonged TpTe likely could increase risk of ventricular arrhythmogenesis [29]. As mentioned above, in the present study barberry extract was associated with increase in QRS interval and decrease in QT interval. The QT interval includes 2 components, depolarization and repolarization, and an increase or decrease in either or both may result in QT prolongation or shortening. Because the JT interval is an independent measure of repolarization [30], we also measured this parameter and observed that it was reduced in presence of high doses of barberry. This effect of barberry extract is similar to the effect of Class IB antiarrhythmic drugs that increase the QRS interval by block of fast sodium channels and decrease of action potential duration, effective refractory period, and hence QT interval by regulating the potassium channels [31].

On the other hand, there is sufficient nonclinical and clinical epidemiological evidence to suggest that shortening of QT interval may be a potential harbinger of proarrhythmia [32]. In addition, the risk of ventricular fibrillation in patients who have a short QTc interval is much higher than normal [33, 34].

Berberine is one of the compounds in barberry and so far some studies reported the antiarrhythmic effect of this agent [35, 36]. Moreover, application of berberine to the current-clamped myocytes produced a significant prolongation of action-potential duration, which was concentration dependent. In the other study, Wang et al. showed that, under the voltage-clamp conditions, berberine inhibited the slowly activating delayed rectifier K^+^ currents, increased the Na^+^-Ca^2+^ exchange currents and the L-type Ca^2+^ currents, and produced a significant prolongation of action-potential duration myocytes. This study suggests that the prolongation of cardiac repolarization by berberine is mainly caused by the inhibition of IKs and increase of ICa [37]. Therefore, based on Wang and Zheng study, unlike the effect of barberry extract on ECG that was observed in the present study, berberine can increase JT, QT, and TpTe intervals through prolongation of cardiac repolarizations.

Comparison of our findings and Wang et al. study strengthens the possibility that berberine is not dominant compound of barberry fruit which is responsible for its effects on ECG. It seems that the other compounds of barberry fruit than berberine are responsible in creating these ECG alterations.

## 5. Conclusion

The findings demonstrate that 2-week pretreatment with aqueous extract of* B. integerrima* fruit extract in doses of 100 and 200 mg/kg/daily increased the depolarization phase and reduced the repolarization phase of ventricular muscle in rat. This effect could potentially be antiarrhythmic and in some conditions could be arrhythmogenesis. The findings also suggest that compounds other than berberine contribute in the effects of barberry fruit extract on ECG and ventricular conductivity.

## Figures and Tables

**Table 1:** Blood pressure, heart rate, and some ECG parameters of animals groups.

Groups	Systolic BP (mmHg)	Diastolic BP (mmHg)	Heart rate (beat/min)	RR interval (ms)	PR interval (ms)	P duration (ms)	Q amplitude (mv)
CTL	119 ± 7	94 ± 5	383 ± 4	157 ± 3	42 ± 2	18 ± 2	−0.021 ± 0.095
B50	125 ± 7	97 ± 5	368 ± 5	164 ± 4	49 ± 2	18 ± 2	−0.016 ± 0.197
B100	111 ± 5	90 ± 3	345 ± 15	175 ± 8	51 ± 2	21 ± 1	−0.029 ± 0.154
B200	111 ± 4	90 ± 3	371 ± 6	163 ± 4	47 ± 3	19 ± 2	−0.013 ± 0.177

Values are mean ± SEM. CTL: control; B50: animal group which received 50 mg/kg/day of barberry extract; B100: animal group which received 100 mg/kg/day of barberry extract; B200: animal group which received 200 mg/kg/day of barberry extract; BP: blood pressure, *n* = 7-8. Values were not significant among groups.
